# Cholinergic white matter pathways in dementia with Lewy bodies and Alzheimer’s disease

**DOI:** 10.1093/brain/awab372

**Published:** 2021-10-04

**Authors:** Julia Schumacher, Nicola J Ray, Calum A Hamilton, Paul C Donaghy, Michael Firbank, Gemma Roberts, Louise Allan, Rory Durcan, Nicola Barnett, John T O’Brien, John-Paul Taylor, Alan J Thomas

**Affiliations:** 1 Translational and Clinical Research Institute, Faculty of Medical Sciences, Newcastle University, Campus for Ageing and Vitality, Newcastle upon Tyne NE4 5PL, UK; 2 Health, Psychology and Communities Research Centre, Department of Psychology, Manchester Metropolitan University, Manchester, UK; 3 Institute of Health Research, University of Exeter, Exeter, UK; 4 Department of Psychiatry, University of Cambridge School of Medicine, Cambridge CB2 0SP, UK

**Keywords:** nucleus basalis of Meynert, basal forebrain, mild cognitive impairment, diffusion-weighted imaging, probabilistic tractography

## Abstract

Patients who have dementia with Lewy bodies and Alzheimer’s disease show early degeneration of the cholinergic nucleus basalis of Meynert. However, how white matter projections between the nucleus basalis of Meynert and the cortex are altered in neurodegenerative disease is unknown.

Tractography of white matter pathways originating from the nucleus basalis of Meynert was performed using diffusion-weighted imaging in 46 patients with Alzheimer’s disease dementia, 48 with dementia with Lewy bodies, 35 with mild cognitive impairment with Alzheimer’s disease, 38 with mild cognitive impairment with Lewy bodies and 71 control participants. Mean diffusivity of the resulting pathways was compared between groups and related to cognition, attention, functional EEG changes and dementia conversion in the mild cognitive impairment groups.

We successfully tracked a medial and a lateral pathway from the nucleus basalis of Meynert. Mean diffusivity of the lateral pathway was higher in both dementia and mild cognitive impairment groups than controls (all *P* < 0.03). In the patient groups, increased mean diffusivity of this pathway was related to more impaired global cognition (β = −0.22, *P* = 0.06) and worse performance on an attention task (β = 0.30, *P* = 0.03). In patients with mild cognitive impairment, loss of integrity of both nucleus basalis of Meynert pathways was associated with increased risk of dementia progression [hazard ratio (95% confidence interval), medial pathway: 2.51 (1.24–5.09); lateral pathway: 2.54 (1.24–5.19)]. Nucleus basalis of Meynert volume was reduced in all clinical groups compared to controls (all *P* < 0.001), but contributed less strongly to cognitive impairment and was not associated with attention or dementia conversion. EEG slowing in the patient groups as assessed by a decrease in dominant frequency was associated with smaller nucleus basalis of Meynert volumes (β = 0.22, *P* = 0.02) and increased mean diffusivity of the lateral pathway (β = −0.47, *P* = 0.003).

We show that degeneration of the cholinergic nucleus basalis of Meynert in Alzheimer’s disease and dementia with Lewy bodies is accompanied by an early reduction in integrity of white matter projections that originate from this structure. This is more strongly associated with cognition and attention than the volume of the nucleus basalis of Meynert itself and might be an early indicator of increased risk of dementia conversion in people with mild cognitive impairment.

## Teaser

Schumacher *et al.* reveal an early reduction in the integrity of cholinergic white matter pathways in Alzheimer’s disease and dementia with Lewy bodies, which is related to global cognitive impairment and attentional deficits, and to conversion to dementia in people with mild cognitive impairment.

## Introduction

Dementia with Lewy bodies (DLB) and Alzheimer’s disease are both characterized by marked cholinergic deficits.^1^ Previous *in vivo* studies of the cholinergic system in these conditions have investigated volumetric changes within the nucleus basalis of Meynert (NBM), a cortically projecting and predominantly cholinergic nucleus located in the basal forebrain.^2-4^ A reduction in NBM volume is evident in both Alzheimer’s disease and DLB patients compared to healthy controls,^5–7^ is already present in people with mild cognitive impairment (MCI),^8–12^ and is associated with global cognitive impairment^7,12^ and impaired performance on attention tasks.^13,14^ Furthermore, the cholinergic system has been implicated in the core Lewy body symptoms of visual hallucinations^15^ and cognitive fluctuations.^5^ Cholinergic remediation with cholinesterase inhibitors is therefore a mainstay of symptomatic treatment in DLB and Alzheimer’s disease.^16,17^

Previous studies have shown that cholinergic projections from the NBM to the cortex travel in discrete, organized bundles.^18^ Two main white matter pathways have been identified that originate from the NBM and pass to different parts of the cortex: A medial pathway travelling through the cingulum towards cingulate, retrosplenial and subcallosal cortex, and a lateral pathway travelling through the external capsule and uncinate fasciculus to innervate the insula as well as frontal, parietal and temporal cortex.^18–20^ Recently, it has been demonstrated that structural integrity of these NBM white matter pathways may make a stronger contribution to cognitive health in normal ageing than NBM volume.^19^ However, whether they also play a role in neurodegenerative diseases like DLB and Alzheimer’s disease is not currently known.

Additionally, our recent work has shown that changes in NBM volume are related to functional EEG changes, indicating that the slowing of the resting-state EEG rhythm that is typically observed in dementia and MCI patients is related to a loss of NBM volume^12^; however, the role of NBM white matter projections in this process has not been investigated yet.

The aim of the present study was therefore to investigate the integrity of the NBM pathways in people with Alzheimer’s disease dementia and DLB compared to age-matched controls without dementia using diffusion-weighted imaging data. In addition to those with an established dementia diagnosis, we also included people at the MCI stage with the aim of assessing whether any changes in NBM pathways occur early in the course of the disease and whether these changes are predictive of future dementia onset in these patients. Finally, we aimed to investigate the contribution of structural integrity of the two NBM pathways described before to decline in global cognitive performance, attention and dementia-related functional EEG changes.

## Materials and methods

### Participants

This analysis included 246 participants from previous studies who were over 60 years of age.^12,21,22^ Fifty-two were diagnosed with probable DLB, 46 with probable Alzheimer’s disease dementia, 38 with probable MCI with Lewy bodies (MCI-LB), 36 with MCI due to Alzheimer’s disease (MCI-AD) and 74 were healthy control participants with no history of psychiatric or neurological illness. Patients were recruited from the local community-dwelling population who had been referred to old-age psychiatry and neurology services. Dementia and MCI diagnoses were performed independently by a consensus panel of three experienced clinicians in accordance with consensus clinical criteria for probable DLB,^23^ probable Alzheimer’s disease dementia,^24^ probable MCI-LB^25^ and MCI-AD.^26^ All MCI participants underwent dopaminergic imaging with FP-CIT SPECT and MIBG myocardial scintigraphy. These scans were used for the diagnosis of probable MCI-LB as set out in the diagnostic criteria^25^ and all participants diagnosed with MCI-AD showed normal MIGB and FP-CIT scans. Healthy control participants were recruited from a local research register and from relatives and friends of patients.

### Neuropsychological testing

All participants underwent a detailed clinical assessment including the Mini-Mental State Examination (MMSE) as a measure of global cognition, the Unified Parkinson’s Disease Rating Scale (UPDRS) part III for the assessment of Parkinsonian motor problems, the Neuropsychiatric Inventory (NPI) and the Clinician Assessment of Fluctuations (CAF) for the assessment of cognitive fluctuations. Participants also performed computerized tests including a choice reaction time task.^27,28^ MCI participants were followed up annually and reassessed with neuropsychological testing and clinical panel review of diagnosis and assessment for the presence of incident dementia.^29^

The study was approved by Newcastle & North Tyneside 1 Research Ethics Committee (10/H0906/19) and Newcastle & North Tyneside 2 Research Ethics Committee (15/NE/0420 and 13/NE/0064), and written informed consent was obtained from all participants.

### MRI acquisition

T_1_-weighted MR images were acquired on a 3 T Philips Intera Achieva scanner with a magnetization prepared rapid gradient echo sequence, sagittal acquisition, echo time 4.6 ms, repetition time 8.3 ms, inversion time 1250 ms, flip angle = 8°, SENSE factor = 2 and in-plane field of view 240 × 240 mm^2^ with slice thickness 1.0 mm, yielding a voxel size of 1.0 × 1.0 × 1.0 mm^3^.

Diffusion-weighted images (DWI) were acquired with the following parameters: repetition time 6126 ms, echo time 70 ms, 124 × 120 matrix; 270 × 270 field of view, 59 slices with slice thickness 2.11 mm, 64 gradient orientations (*b* = 1000 s/mm^2^) and six images without diffusion weighting (*b* = 0 s/mm^2^, b0).

### MRI preprocessing

The methods below approximately follow the pipeline developed in Nemy *et al*.^19^ for NBM segmentation and diffusion-based tracking of NBM white matter projections.

**Figure 1:**
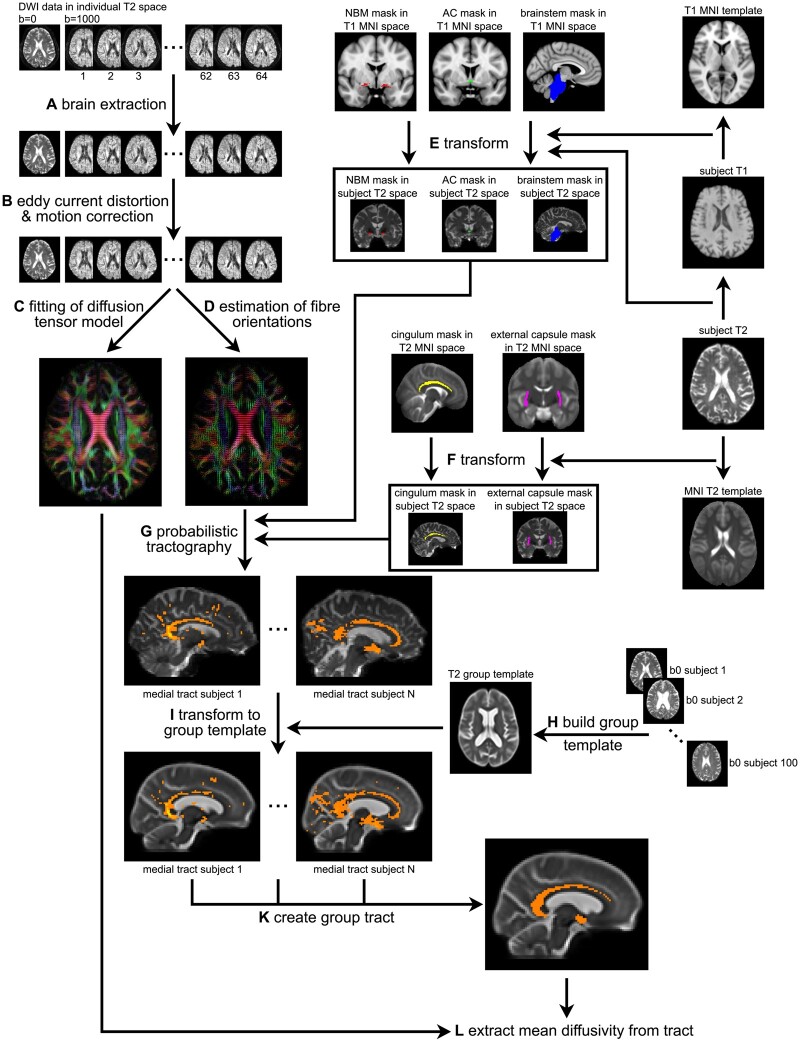
**Overview of NBM white matter tract estimation.** (**A**) Brain extraction of diffusion-weighted data using FSL’s bet function. (**B**) Correction for eddy currents and head motion using FSL’s eddy tool. (**C**) Fitting of the diffusion tensor model using FSL’s dtifit. (**D**) Estimation of fibre orientations in each voxel using FSL’s BedpostX with a ball-and-stick model with three fibres per voxel. (**E**) Transformation of region of interest from T_1_ MNI space to individual subject T_2_ space. (**F**) Transformation of region of interest from standard T_2_ space to individual subject T_2_ space. (**G**) Probabilistic tractography with FSL’s ProbtrackX using the NBM region of interest as seed, the cingulum and external capsule region of interest as waypoint masks, and the brainstem and anterior commissure as exclusion masks. (**H**) Building of unbiased group template from 100 randomly selected b0 images. (**I**) Transformation of tracts from individual subject T_2_ space to the unbiased group template. (**K**) Estimation of group tracts for the medial and lateral NBM pathways. (**L**) Extraction of diffusion parameters from the estimated tracts. AC = anterior commissure.

The DWI data were visually inspected for artefacts and then processed using the FSL toolbox (v.6.0.2). As a first step, brain extraction was performed using FSL’s bet function^30^ (Fig. 1A). Second, FSL’s eddy tool was applied to correct for eddy currents and head motion^31^ (Fig. 1B). Participants whose average absolute motion exceeded 3 mm were excluded from further analysis.

FSL’s dtifit was then applied to fit a diffusion tensor model to the data (Fig. 1C) and BedpostX was applied to calculate diffusion parameters using a standard ball-and-sticks model in each voxel with three fibres modelled per voxel^32^ (Fig. 1D).

### Regions of interest

Five region of interest masks were used to guide the probabilistic tractography based on previous research.^18–20^ A mask of the NBM was used as the seed region for the tractography. This was based on a cytoarchitectonic map of the cholinergic basal forebrain in MNI T_1_ space that has been derived from combined histology and MRI of a post-mortem brain.^33^ The NBM region of interest was created by combining the anterior-lateral, anterior-intermediate and posterior Ch4 subregions of the basal forebrain map. Previous studies have identified two main white matter pathways originating from the NBM: a medial pathway that passes through the cingulum and a lateral pathway going through the external capsule.^18,19^ Regions of interest of the cingulum and external capsule were therefore used as waypoint masks to constrain the tractography to these pathways. The cingulum and external capsule masks were obtained from the Johns Hopkins University white matter atlas in FSL^34^ in standard T_2_ space. Finally, regions of interest of the anterior commissure and the brainstem were used as exclusion masks to avoid contamination of estimated tracts from non-cholinergic pathways.^4,19^ The anterior commissure mask was obtained from FSLs’ XTRACT tool^35^ in MNI T_1_ space and the brainstem mask was estimated using FSL’s FIRST segmentation routine.^36^

All five regions of interest were transformed into each individual’s T_2_ space. Subject-space T_1_ images were transformed to MNI T_1_ space and each subject’s T_2_ (b0) image was registered with the corresponding T_1_ image. The inverse transforms of these two registration steps were then applied to warp the regions of interest that were defined in MNI T_1_ space (NBM, anterior commissure, brainstem) to subject-specific T_2_ space (Fig. 1E). Transformations from each subject’s T_2_ image to the standard DTI template were estimated and the inverse transformations were applied to register regions of interest from standard DTI space (cingulum, external capsule) to subject T2 space (Fig. 1F). All transformations were estimated with Advanced Normalization Tools (ANTs),^37^ using ANTs’ non-linear SyN registration algorithm for subject T_1_ → MNI T_1_ and subject T_2_ → standard T_2_ space and affine registration between subject T_1_ and T_2_ images.

### Tractography

Probabilistic tracking was performed with FSL’s ProbtrackX by generating 5000 random samples from the NBM seed region of interest that propagated through the local probability density function of the estimated diffusion parameters^38^ (Fig. 1G). The cingulum and external capsule regions of interest were used as waypoint masks, i.e. only streamlines that passed through either of these regions of interest were retained. Pathways that entered the brainstem or anterior commissure were excluded.

Tracts estimated in individual subject space were brought to a common space to estimate a group tract. An unbiased group template was created with ANTs’ buildtemplate function using the b0 images of 100 randomly selected participants (20 from each diagnostic group, Fig 1H), and individual subject tracts were transformed to the group template space (Fig. 1J). Medial and lateral group tracts were created by including all voxels that were included in at least 50% of the individual subject tracts (Fig. 1K). This threshold was chosen based on visual inspection of the resulting group tracts and is lower than in Nemy *et al*.,^19^ potentially due to greater atrophy in our patient populations. The medial and lateral group tracts were then transformed back into individual subject T_2_ space and mean diffusivity from voxels belonging to each tract was extracted for each participant (Fig. 1L).

The analyses presented here were focussed on mean diffusivity based on the previous study by Nemy *et al*.^19^ and due to the fact that mean diffusivity appears to be an earlier marker of white matter changes in neurodegenerative disease compared to other DTI metrics such as fractional anisotropy.^39–41^ As a supplementary analysis, we also investigated radial and axial diffusivity.

To test the specificity of any effects for the NBM white matter pathways, we created a white matter control mask by subtracting the two NBM tracts from a whole-brain white matter mask that was obtained by running FSL FAST on each subject’s T_1_-weighted image.^42^

### Estimation of NBM volumes

Preprocessing of T_1_-weighted MR images was performed in SPM12 (http://www.fil.ion.ucl.ac.uk/spm/). Images were first segmented into grey matter, white matter and cerebrospinal fluid. The DARTEL algorithm was used to create a study-specific template and all grey matter images were coregistered to this template.^43^ To transform the NBM mask from MNI standard space to the study-specific DARTEL template space, we used ANTs’ non-linear SyN algorithm to register the DARTEL template to MNI space and applied the inverse transform to the NBM map. Mean grey matter volumes within the NBM mask were calculated and proportionally normalized by total intracranial volume.

### EEG acquisition and analysis

Eyes closed resting-state EEG data (128 electrodes) were available for a subset of participants (49 controls, 33 MCI-AD, 25 Alzheimer’s disease dementia, 37 MCI-LB and 20 DLB). Acquisition and preprocessing have been described in detail before.^44^ Briefly, preprocessing included bandpass-filtering between 0.3 and 54 Hz and epoching the data into non-overlapping 2 second epochs. Visually identified noisy channels and epochs were excluded before applying independent component analysis for further removal of muscular, cardiac, ocular and line noise artefacts. Previously excluded channels were then replaced using spherical spline interpolation and data were average referenced. Power spectral density was estimated using Bartlett’s method in MATLAB and the dominant frequency was calculated as the frequency with the highest power between 4 and 15 Hz (averaged across epochs and electrodes).

### Statistics

Statistical analyses were performed in R (https://www.r-project.org/) and figures were produced using the package ggplot2.^45^ Normalized NBM volumes were compared between the groups using a univariate analysis of covariance (ANCOVA) controlling for age and sex, followed by *posthoc* tests with Bonferroni correction for multiple comparisons.

Mean diffusivity from the two NBM tracts was compared between the groups with a univariate ANCOVA controlling for age, sex and mean diffusivity from the white matter control mask. The ANCOVA was followed by pairwise *posthoc* tests with Bonferroni correction.

As an additional control analysis, we also applied univariate ANCOVAs to investigate group differences in mean diffusivity within the standard white matter regions of interest (cingulum and external capsule) that were used to guide the tractography (controlling for age and sex).

We also calculated Pearson’s correlations between NBM volume and mean diffusivity values of the NBM pathways and from the white matter control mask.

Cox proportional hazards models were estimated in R to assess risks of developing dementia in the MCI groups, and associations with NBM volume, and mean diffusivity of the medial and lateral NBM white matter pathways. The latter two predictors were modelled controlling for the mean diffusivity of the white matter control mask. All models also controlled for age at baseline, and cognitive function at baseline as assessed by the Addenbrooke’s Cognitive Examination—Revised. Age was centred at the grand mean, while cognitive function and all NBM volumetric and diffusivity variables were standardized with respect to the control group.

To test the association of NBM volume and the integrity of the NBM tracts with global cognition, a multiple linear regression model was run across all MCI and dementia patients with MMSE as the dependent variable and mean diffusivity along the medial and lateral NBM tracts and NBM volume as predictor variables, including age, sex, diagnosis and mean diffusivity from the white matter control mask as covariates. A similar model was run with mean choice reaction time as the dependent variable to test the association with attentional performance and with EEG dominant frequency as the dependent variable to test the relationship with EEG slowing.

Additionally, we tested the degree of contribution of NBM volume and NBM tract integrity to cognitive measures and EEG slowing by performing a random forest regression analysis with conditional inference trees for unbiased variable selection using the party package in R.^46,47^ Random forests are an ensemble learning method that can be used to determine prediction accuracy. The relative importance of a feature for the overall prediction can be assessed by a conditional feature importance score that is computed by measuring the increase in prediction error (i.e. mean squared error between predicted and actual response) when permuting the feature of interest on a grid defined by the other features included in the model. This analysis was run separately for MMSE, choice reaction time and EEG slowing including features for mean diffusivity along the medial and lateral NBM tracts, normalized NBM volume, mean diffusivity of the white matter control mask, age and sex. The number of trees was set to 3000.

To investigate associations with clinical symptoms in the Lewy body groups, we used multiple linear regression with CAF scores, NPI hallucination scores and UPDRS scores as dependent variables including mean diffusivity along the lateral and medial NBM tracts, NBM volume and mean diffusivity from the white matter control mask as predictors.

Finally, to test whether the use of cholinesterase inhibitors was associated with NBM tract integrity or NBM volumes, we compared NBM volume and mean diffusivity along the medial and lateral NBM tracts between patients taking cholinesterase inhibitors (*n* = 108) and patients not taking these medications (*n* = 56) using univariate ANCOVAs including a covariate for cognitive status (MCI/dementia) to control for the fact that more dementia patients were taking cholinesterase inhibitors compared to the MCI groups.

### Data availability

The data that support the findings of this study are available from the corresponding author, on reasonable request.

## Results

### Demographics

Three DLB participants were excluded because of artefacts on the diffusion-weighted data. Additionally, three controls, one patient with DLB and one MCI-AD patient were excluded because of excessive motion. Thus, 35 participants with MCI-AD, 46 with Alzheimer’s disease dementia, 38 with MCI-LB, 48 with DLB and 71 healthy controls were included for further analysis. There was no difference in average motion between the groups [*F*(4,233) = 1.6, *P* = 0.2]. Table 1 shows an overview of demographics and clinical information. As expected, global cognition as measured by the MMSE was more impaired in the two dementia groups compared to the MCI groups, but there was no difference between MCI-AD and MCI-LB or between Alzheimer’s disease dementia and DLB.

**Table 1:** Demographics and clinical information

	Controls (*n* = 71)	MCI-AD (*n* = 35)	MCI-LB (*n* = 38)	AD (*n* = 46)	DLB (*n* = 48)	Group differences
Male:female	51:20	15:20	34:4	38:8	35:13	χ^2^ = 23.4, *P* < 0.001^a^
						*P*(HC, MCI-AD) = 0.004
						*P*(HC, MCI-LB) = 0.034
						*P*(MCI-AD, MCI-LB) < 0.001
						*P*(MCI-AD, AD) < 0.001
						*P*(MCI-AD, DLB) = 0.006
						*P*(MCI-LB, DLB) = 0.06
Age	75.3 (6.9)	75.8 (7.8)	74.5 (6.5)	77.1 (7.5)	76.2 (6.6)	*F*(4,233) = 0.83, *P* = 0.51^b^
AChEI	–	7 (21%)^e^	18 (49%)^f^	39 (85%)	44 (92%)	χ^2^ = 55.7, *P* < 0.001^c^
						*P*(MCI-AD, MCI-LB) = 0.02
						*P*(MCI-AD, AD)<0.001
						*P*(MCI-AD, DLB)<0.001
						*P*(MCI-LB, AD)<0.001
						*P*(MCI-LB, DLB)<0.001
PD meds	–	0^e^	4 (11%)^f^	0	21 (44%)	χ2 = 45.0, *P* < 0.001^c^
						*P*(MCI-AD, MCI-LB) = 0.052
						*P*(MCI-AD, DLB) < 0.001
						*P*(MCI-LB, AD) = 0.02
						*P*(MCI-LB, DLB) = 0.001
						*P*(AD, DLB)<0.001
MMSE	28.8 (1.0)	26.8 (2.1)	26.5 (2.4)	21.1 (4.0)	22.6 (4.5)	*F*(3,163) = 26.0, *P* < 0.001^d^
						*P*(MCI-AD, AD) < 0.001
						*P*(MCI-AD, DLB) < 0.001
						*P*(MCI-LB, AD) < 0.001
						*P*(MCI-LB, DLB) < 0.001
UPDRS III	–	8.3 (7.3)	12.3 (7.6)	3.6 (2.7)	19.3 (8.8)	*F*(3,162) = 42.3, *P* < 0.001^d^
						*P*(MCI-AD, AD) = 0.02
						*P*(MCI-AD, DLB) < 0.001
						*P*(MCI-LB, AD) < 0.001
						*P*(AD, DLB) < 0.001
CAF total	–	1.4 (2.7)^g^	3.7 (4.3)^h^	1.9 (3.9)^i^	5.0 (4.4)^k^	*F*(3,150) = 6.7, *P* < 0.001^d^
						*P*(MCI-AD, DLB) = 0.002
						*P*(AD, DLB) = 0.002
NPI total	–	8.4 (9.5)^g^	16.0 (13.0)^h^	8.5 (8.7)^i^	14.3 (13.4)^k^	*F*(3,150) = 4.3, *P* = 0.006^d^
						*P*(MCI-AD, MCI-LB) = 0.07
						*P*(MCI-LB, AD) = 0.03
NPI hall	–	0.04 (0.2)^l^	0.6 (1.1)^h^	0.07 (0.4)^i^	2.1 (2.4)^k^	*F*(3,148) = 18.5, *P* < 0.001^d^
						*P*(MCI-AD, DLB) < 0.001
						
						*P*(AD, DLB) < 0.001

ACE-R = Addenbrooke’s Cognitive Examination-Revised; AChEI = number of patients taking acetylcholinesterase inhibitors; AD = Alzheimer’s disease dementia; CAF total = Clinician Assessment of Fluctuation total score; HC = healthy controls; NPI hall = NPI hallucination score; PD meds = number of patients taking dopaminergic medication for the management of Parkinson’s disease symptoms; UPDRS III = Unified Parkinson’s disease Rating Scale III (motor subsection).

^a^ Chi-square test all groups.

^b^ Univariate ANOVA all groups.

^c^ Chi-square test MCI and dementia groups.

^d^ Univariate ANOVA MCI and dementia groups.

^e^ *n* = 33;

^f^ ^f^ *n* = 37;

^g^ ^g^ *n* = 27;

^h^ ^h^ *n* = 35;

^i^ ^i^ *n* = 45;

^j^ ^k^ *n* = 47;

^k^ ^l^ *n* = *25*.

Fifteen MCI-AD and 30 MCI-LB patients had undergone at least one clinical reassessment at the point of data locking (additional data collection delayed due to COVID-19), and a further two had died before any follow-up (excluded from longitudinal analysis). There were 14 cases of dementia identified in the follow-up from MCI cohorts: five from MCI-AD (all clinical Alzheimer’s disease dementia) and nine from MCI-LB (all probable DLB); these occurred after a mean of 1.5 years. The remaining 31 MCI patients had been assessed for a mean of 1.6 years since baseline without developing dementia.

### NBM white matter pathways

**Figure 2:**
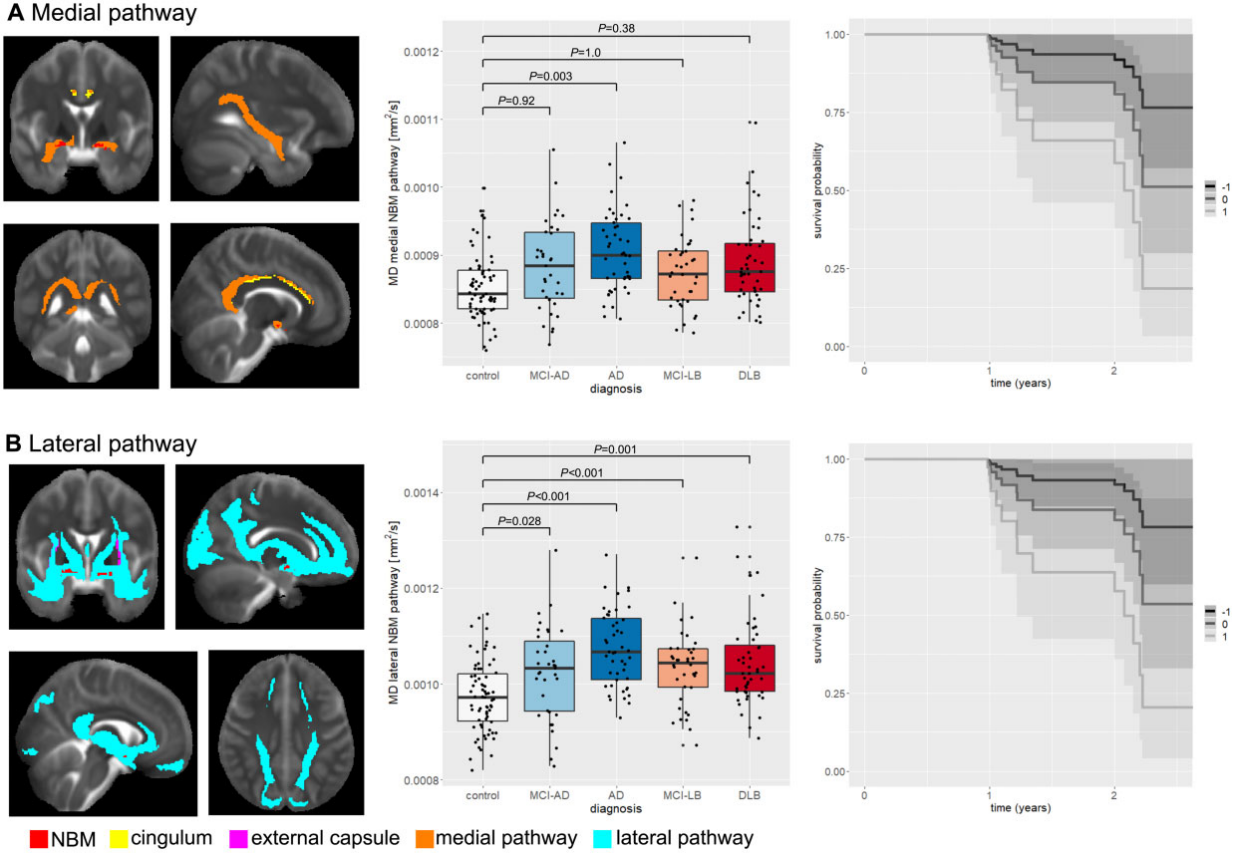
**Group comparison of NBM pathway integrity.** (**A**) Medial NBM pathway (shown in orange) estimated with the NBM region of interest as seed (shown in red), the cingulum as waypoint mask (shown in yellow), and brainstem and anterior commissure as exclusion masks (not shown). (**B**) Lateral NBM pathway (shown in cyan) estimated with the NBM region of interest as seed (shown in red), the external capsule as waypoint mask (shown in pink), and the brainstem and anterior commissure as exclusion masks (not shown). In each box plot the central line corresponds to the sample median, the upper and lower border of the box represent the 25th and 75th percentile, respectively, and the length of the whiskers corresponds to 1.5 times the interquartile range. *P*-values result from pairwise *post hoc* tests with Bonferroni correction for multiple comparisons. All *P*-values for comparisons that are not shown are >0.05. The plots on the right show survival probabilities in MCI without dementia given a standardized (**A**) medial and (**B**) lateral pathway mean diffusivity of −1, 0, or +1 (representative *z*-scores with 0 being the mean, +1 being 1 standard deviation (SD) higher and −1 being 1 SD lower than mean diffusivity). AD = Alzheimer’s disease dementia; MD = mean diffusivity.

The medial and lateral NBM group tracts are shown in Fig. 2A and B. They include comparable white matter regions to those found in previous studies of NBM tractography.^19,20^ The medial pathway projects from the NBM to the cingulum and continues to the cingulate and retrosplenial cortices. The lateral pathway travels through the external capsule and the uncinate fasciculus to the frontal pole and via the posterior thalamic radiation and internal capsule towards parietal and temporal cortex.

### Group comparison of NBM tracts

After controlling for age, sex and mean diffusivity from the white matter control mask, there was an overall effect of diagnosis for mean diffusivity along the medial (cingulum) NBM pathway: *F*(4,230) = 3.4, *P* = 0.01. *Posthoc* tests revealed that mean diffusivity along the medial pathway was increased in Alzheimer’s disease dementia compared to controls (*P* = 0.003), whereas all other pairwise comparisons between both MCI and both dementia groups were not significant (*P* > 0.05, Fig. 2A). When the covariate for mean diffusivity from the white matter control mask was removed from the model, there was a significant increase in mean diffusivity along the medial NBM pathway in Alzheimer’s disease dementia compared to controls (*P* < 0.001) and in DLB patients compared to controls (*P* = 0.009) while all other pairwise comparisons remained non-significant (all *P* > 0.05).

For mean diffusivity in the lateral (external capsule) NBM pathway, the univariate ANCOVA revealed a significant overall effect of diagnosis: *F*(4,230) = 9.9, *P* < 0.001. *Posthoc* tests showed that mean diffusivity along the lateral pathway was increased in both MCI and both dementia groups compared to controls (Fig. 2B) with no significant difference between any of the MCI and dementia groups. These results remained the same when not controlling for mean diffusivity from the white matter control mask.

The supplementary analysis of group differences in axial and radial diffusivity showed very similar results to the analysis of mean diffusivity (Supplementary Table 1).

There was no significant effect of diagnosis on mean diffusivity of the standard cingulum [*F*(4,231) = 2.0, *P* = 0.1] and external capsule regions of interest [*F*(4,231) = 0.9, *P* = 0.5], controlling for age and sex.

### Group comparison of NBM volume

**Figure 3:**
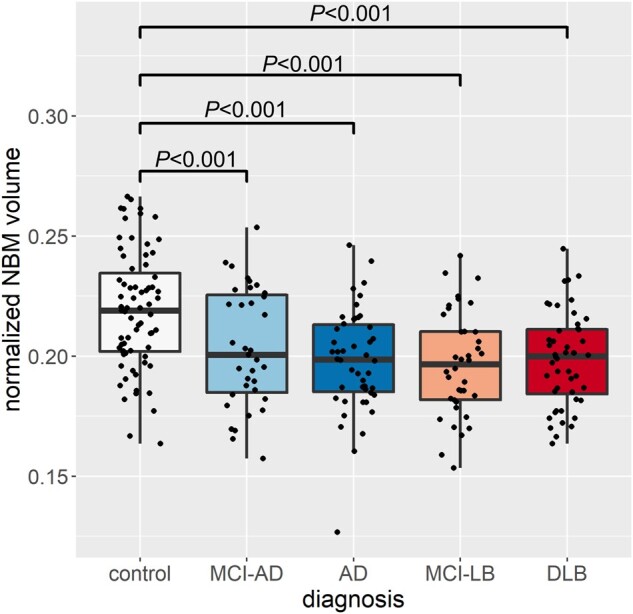
**Group comparison of normalized NBM volume.** In each box plot the central line corresponds to the sample median, the upper and lower border of the box represent the 25th and 75th percentile, respectively, and the length of the whiskers corresponds to 1.5 times the interquartile range. *P*-values result from pairwise *post hoc* tests with Bonferroni correction for multiple comparisons. All *P*-values for comparisons that are not shown are >0.05. AD = Alzheimer’s disease dementia.

There was an effect of diagnosis on normalized overall NBM volumes after controlling for age and sex: *F*(4,231) = 11.0, *P* < 0.001. *Posthoc* tests showed that NBM volume was significantly reduced in both MCI and dementia groups compared to controls with no significant difference between the MCI and dementia groups (Fig. 3).

### Association between NBM volume and pathway integrity

Figure 5 shows scatterplots between NBM volume and mean diffusivity along the two NBM pathways and from the white matter control mask, with Pearson’s correlation values (across all participants and within each diagnostic group separately) shown in the upper right part. Mean diffusivity from the medial and lateral pathways showed relatively high correlation values between 0.70 and 0.88 whereas NBM pathway diffusivity was moderately correlated with NBM volume (between −0.09 and −0.51) and with mean diffusivity of the white matter control mask (values between 0.41 to 0.65).

### Survival analysis

Primary analyses in Table 2 explored whether (i) NBM volume; [ii(a)] mean diffusivity of the medial NBM pathway; and [ii(b)] mean diffusivity of the lateral NBM pathway were associated with increased risk of dementia onset. Normalized NBM volume was not a significant positive or negative predictor of dementia onset in MCI. In both the medial and lateral NBM white matter tracts, higher mean diffusivity was associated with significantly increased risk of dementia onset (Fig. 2). These results survived correction for diagnostic group, but not cholinesterase inhibitor use (Table 2).

**Table 2:** Primary analysis of NBM volume and mean diffusivity of NBM pathways predicting dementia onset, and sensitivity analyses controlling for diagnostic group differences and cholinesterase inhibitor use

Predictor^a^	Primary analysis	Sensitivity analyses	
		Diagnostic group	Cholinesterase inhibitor use
Normalized NBM volume	0.97 [0.55–1.69]	–	–
Mean diffusivity, NBM medial pathway^b^	2.51 [1.24–5.09]	2.50 [1.23–5.07]	2.07 [0.88–4.89]
Mean diffusivity, NBM lateral pathway^b^	2.54 [1.24–5.19]	2.95 [1.31–6.64]	1.54 [0.62–3.83]

Data are presented as hazard Ratio [95% CI].

^a^ All controlling for baseline age and cognitive function.

^b^ Also controlling for mean diffusivity of the whiter matter control mask.

Correlations between Schoenfeld residuals and time were assessed for each included variable in each model; in each case no significant associations were identified, supporting the assumption of proportional hazards.

### Association with cognitive performance and EEG slowing

**Figure 4:**
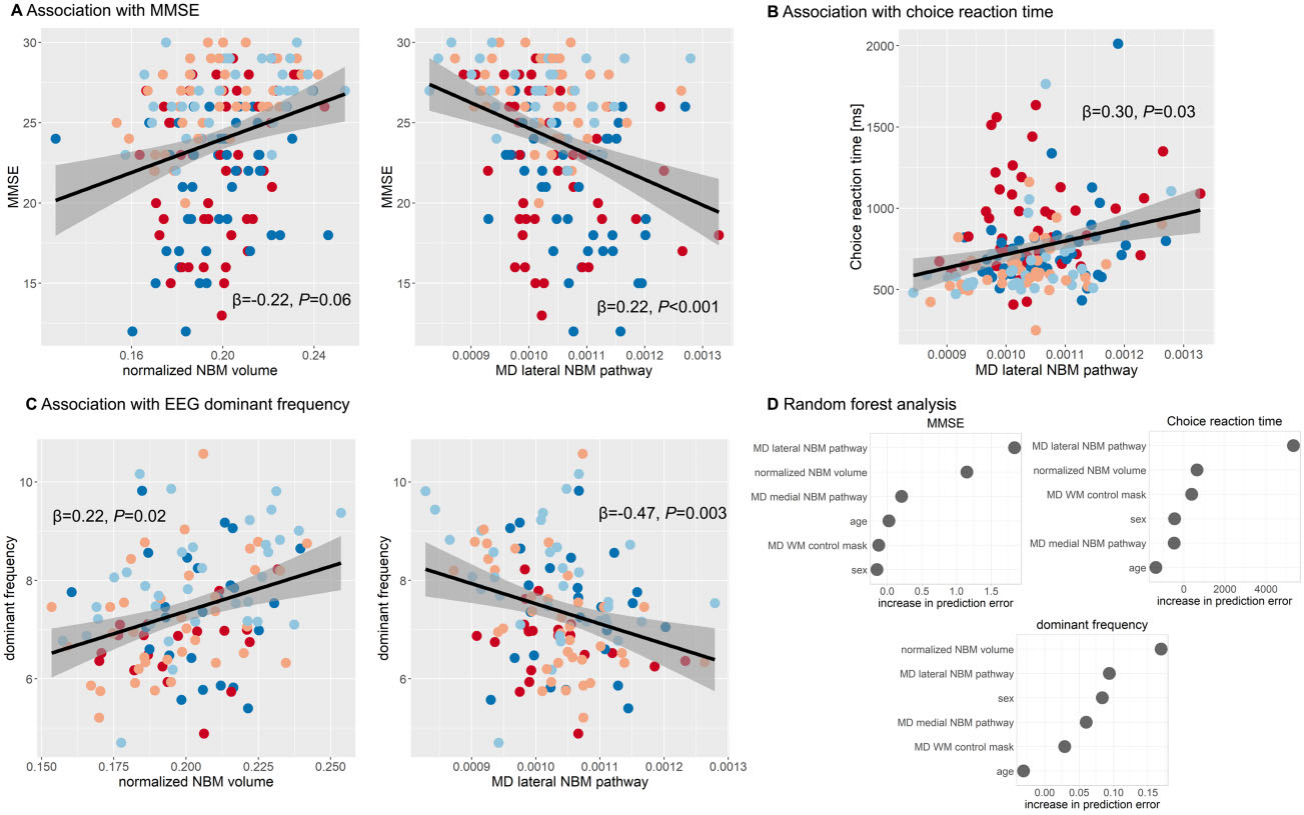
**Association with cognitive and attentional performance.** Association between mean diffusivity (MD) along NBM pathways and (**A**) MMSE as a measure of overall cognition, (**B**) choice reaction time and (**C**) EEG dominant frequency. β-values result from a multiple linear regression analysis including mean diffusivity along the two NBM pathways, normalized NBM volume, diagnosis, age, sex and mean diffusivity from the white matter control mask as predictors. Grey shaded areas correspond to 95% confidence intervals. (**D**) Results from the random forest analysis showing the conditional feature importance (i.e. increase in prediction error when permuting a feature on a grid defined by the other features in the model). Higher values indicate higher importance of a feature in predicting the response variable (choice reaction time, MMSE and EEG dominant frequency). AD = Alzheimer’s disease dementia; MD = mean diffusivity; WM = white matter.

Only normalized NBM volume (β = 0.22, *P* < 0.001) and mean diffusivity of the lateral NBM tract (β = −0.22, *P* = 0.06) uniquely predicted MMSE scores beyond age (Fig. 4A), while mean diffusivity along the medial tract (β = 0.04, *P* = 0.73) and mean diffusivity from the white matter control mask (β = 0.04, *P* = 0.59) did not.

**Figure 5:**
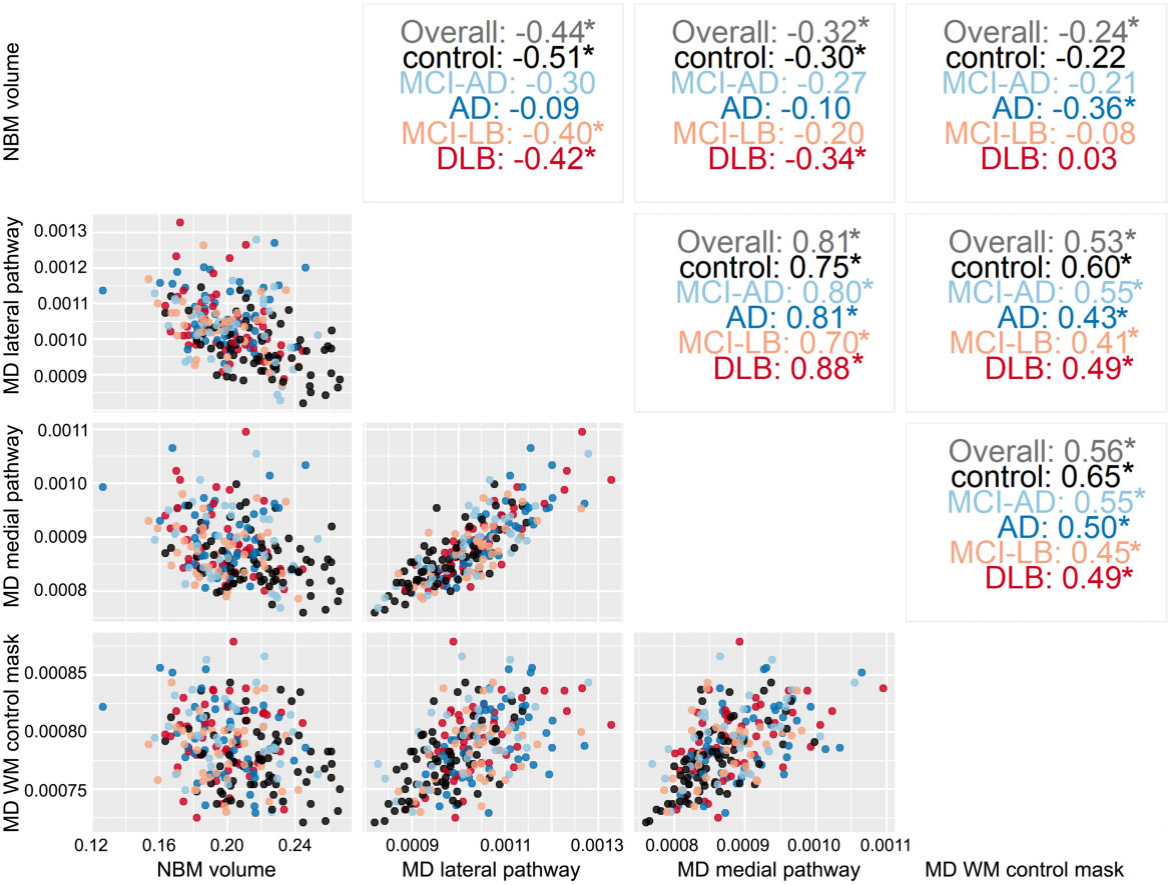
**Correlation structure between different predictor variables.** Scatterplots (*bottom left*) and Pearson’s correlation values (*top right*) between mean diffusivity from the two NBM pathways, NBM volume and diffusivity of the white matter control mask, across all diagnostic groups and for each group separately. Significant correlations (*P* < 0.05) are marked with an asterisk. AD = Alzheimer’s disease dementia; MD = mean diffusivity; WM = white matter.

Choice reaction time (note that data were available for 32 patients with MCI-AD, 45 with Alzheimer’s disease dementia, 37 with MCI-LB and 47 with DLB) was uniquely predicted by mean diffusivity of the lateral NBM tract (β = 0.30, *P* = 0.03, Fig. 4B) while normalized NBM volume (β = −0.09, *P* = 0.22), mean diffusivity of the medial tract (β = −0.10, *P* = 0.44) and mean diffusivity of the white matter control mask (β = 0.03, *P* = 0.76) did not contribute significantly to the model.

EEG dominant frequency was significantly predicted by mean diffusivity of the lateral NBM tract (β = −0.47, *P* = 0.003) and NBM volume (β = 0.22, *P* = 0.01, Fig. 4C), whereas mean diffusivity of the medial tract (β = 0.22, *P* = 0.12) and of the white matter control mask (β = 0.0, *P* = 0.99) were not significant predictors.

The random forest analysis confirmed these results: mean diffusivity of the lateral NBM tract achieved the strongest importance score in the prediction of both MMSE and choice reaction time performance and also contributed to the prediction of EEG dominant frequency (Fig. 4D). Normalized NBM volume contributed to the prediction of MMSE and EEG dominant frequency whereas all other features received relatively low conditional feature importance scores.

### Association with clinical symptoms in the Lewy body groups

The regression models predicting Lewy body symptom scores from NBM tract integrity and NBM volume were not significant for CAF [*F*(4,76) = 0.87, *P* = 0.49] and NPI hallucination score [*F*(4,77) = 0.96, *P* = 0.43], whereas UPDRS score was associated with NBM volume (β = 0.28, *P* = 0.009), with mean diffusivity of the lateral NBM tract (β = 0.46, *P* = 0.01), and with mean diffusivity from the white matter control mask (β = −0.34, *P* = 0.004).

There was no difference between participants taking cholinesterase inhibitors and those not taking these medications after controlling for cognitive status for mean diffusivity of the lateral NBM tract [*F*(1,161) = 0.2, *P* = 0.67] or NBM volume [*F*(1,161) = 1.0, *P* = 0.3], whereas mean diffusivity of the medial NBM tract was slightly higher in participants who were not taking cholinesterase inhibitors [*F*(1,161) = 3.9, *P* = 0.05].

## Discussion

In this study, we investigated the integrity of white matter pathways originating from the NBM in Alzheimer’s disease dementia and DLB, and their respective MCI stages using diffusion-weighted imaging. We found reduced integrity of the lateral NBM pathway in both dementia and MCI groups compared to controls. In the patient groups, integrity of this pathway was related to global cognition, attentional performance and EEG slowing, and a loss of integrity of both NBM pathways was associated with an increased risk of progression to dementia in MCI. While NBM volume was also reduced in all clinical groups compared to controls, this contributed less strongly to global cognitive impairment and was not associated with attentional performance or conversion to dementia. Finally, the inclusion of mean diffusivity from a white matter control mask in our analyses confirmed these results were specific to NBM white matter tracts.

Degeneration of the NBM is a hallmark of Alzheimer’s disease and DLB, and previous studies have shown that atrophy of this region occurs early in both conditions.^1^ Post-mortem and PET studies have described a cortical cholinergic depletion in people with dementia that has been suggested to be mainly due to degeneration of cholinergic neurons within the NBM.^48–50^ However, the white matter pathways originating from the NBM that provide cortical cholinergic innervation are largely unexplored in the context of neurodegenerative dementia.

We successfully tracked the two main NBM white matter pathways as previously described in immunohistochemical^18^ and DTI tractography studies.^19,20^ We found impaired white matter microstructure within the lateral (external capsule) pathway in both MCI and dementia groups compared to controls. In contrast, the medial (cingulum) pathway was only affected in people with Alzheimer’s disease dementia whereas changes in this pathway in the DLB group did not go beyond global diffusivity changes. Previous post-mortem work has suggested that degeneration of neurons within the NBM leads to a destruction of axons that are travelling from this structure to the cortical surface.^51^ This degenerative process within the NBM is thought to be mainly driven by neurofibrillary tangle deposition and the presence of Lewy bodies rather than amyloid deposition.^51,52^ Furthermore, a relationship between Lewy body pathology in the NBM and the cortical cholinergic deficit has been established,^48^ suggesting that Lewy body pathology is also associated with the degeneration of ascending NBM pathways. The involvement of both Alzheimer’s disease and Lewy body pathology in NBM degeneration is consistent with our observation that changes in the NBM white matter pathways occurred in both MCI and dementia groups. We did not observe any significant differences in the pairwise comparison between the Alzheimer’s disease and Lewy body groups, indicating that changes in NBM pathways present similarly across the two conditions. While this comparison might have been underpowered to detect small group differences, our sample size was relatively large compared to other studies, and the lack of differences between the two dementia groups is in line with previous findings indicating that cholinergic deficits are involved in both conditions.^53,54^

Previous studies in healthy individuals and people with vascular cognitive impairment have reported an association between NBM pathway integrity and white matter lesion load, indicating that vascular disease might also be related to a loss of integrity in cholinergic pathways.^19,20^ However, it has been shown that the presence of white matter hyperintensities in people with neurodegenerative dementia might indicate cortical Alzheimer’s disease pathology rather than vascular disease,^55,56^ suggesting that the influence of vascular processes on the observed NBM pathway alterations in the present study might be less substantial. Nevertheless, studying the impact of the different Alzheimer’s disease- and Lewy body-associated pathologies and vascular factors on NBM pathway integrity will be an important aspect of future studies.

The severity of global cognitive impairment in the patient groups was associated with a loss of integrity of the lateral NBM pathway and reduced NBM volumes, consistent with previous studies.^7,12,14^ In contrast, attentional performance was only associated with NBM pathway integrity, but not with NBM volume. This was confirmed in the random forest analysis, which indicated that mean diffusivity of the lateral NBM pathway was a stronger contributor to global cognitive and choice reaction time performance than NBM volumes. This is in line with a previous study in healthy individuals that found a stronger contribution of the NBM white matter pathways to cognitive performance compared to NBM volume.^19^ Moreover, we found that a greater loss of integrity of both NBM pathways was related to a higher risk of dementia conversion in people with MCI, whereas NBM volume was not. Taken together, these results underline the importance of studying other aspects of the cholinergic system besides NBM volumes—which has been typically done as the standard until now—and suggest that in Alzheimer’s disease and DLB, the integrity of the ascending NBM white matter pathways might be more directly related to a loss of cognitive function and dementia progression. However, in contrast to the present findings, previous studies have found NBM volume to be a predictor of future cognitive decline in Parkinson’s disease^39,57^ and Alzheimer’s disease.^11^ The lack of association in our study might be explained by the relatively short follow-up time of our MCI cohort. Further longitudinal follow-up data are therefore needed to further elucidate the relationship between NBM degeneration and cognitive decline in MCI-LB.

EEG slowing as measured by dominant frequency was related to NBM volume and integrity of the lateral, but not the medial NBM pathway, mirroring the regression results for the cognitive measures. These results are in line with previous findings in dementia suggesting a relationship between EEG slowing and the cholinergic system.^12,58,59^ We extend these by showing that not only the volume of the NBM itself, but also the integrity of its cortical connections is associated with EEG slowing.

### Limitations

This study is subject to some limitations. First, some patients were taking cholinesterase inhibitors, which have been shown to influence NBM degeneration.^60^ The effect of cholinesterase inhibitors on the integrity of NBM white matter projections has not yet been investigated and we did not find differences between patients who were taking cholinesterase inhibitors and those who were not taking this medication. For the survival analysis we found no significant effects while controlling for cholinesterase inhibitor use. However, this may reflect a post-treatment bias since cholinesterase inhibitors were not randomly allocated through this observational study, i.e. those who were developing dementia were more likely to be prescribed these medications. The prescription rates of cholinesterase inhibitors in this patient sample reflect local prescription practices and follow recent guidelines.^16^ Nevertheless, future studies should investigate the effect of this medication on NBM pathways in a prospective manner as well. A second limitation is the fact that our groups were not balanced with respect to sex due to the differences in prevalence of Alzheimer’s disease and DLB in males and females.^61,62^ This might have influenced the analysis given that there is some evidence for sex differences in NBM structure.^63^ We have included sex as a covariate to minimize the influence of this imbalance on the reported group differences. Nevertheless, studying the influence of sex on NBM white matter pathways in people with dementia and MCI might be an interesting avenue for future studies.

For the survival analysis, the number of MCI cases with follow-up data was small, with a relatively short follow-up time. While the incidence of dementia was quite high compared to other prospective studies in MCI, there were few cases overall. While our tests did not refute the assumption of proportional hazards, this could be due to the small sample conferring low power to these tests. In both cases, a larger sample and longer assessment period are required to validate these findings.

Another potential limitation is the lack of Alzheimer’s disease biomarkers in this study.

While we successfully identified the two NBM pathways that have been described in previous studies,^18–20^ the actual cholinergic contribution to these tracts cannot be resolved with diffusion MRI data. The similarity with tracts described in a previous immunohistochemistry study with cholinergic markers^18^ provides some evidence for their cholinergic nature. Additionally, our control analysis of mean diffusivity within the standard cingulum and external capsule regions of interest did not show any significant group differences, indicating that the observed changes are more specific to the reconstructed NBM pathways and not merely a reflection of general diffusivity changes within the anatomically defined fibre bundles that were used to guide the tractography.

## Conclusion

The present study sheds important new light on the integrity of the cholinergic system in neurodegenerative dementia. We extend previous findings by showing that the well-established observation of degeneration of the NBM itself is accompanied by a reduction in the integrity of white matter pathways originating from this structure in Alzheimer’s disease dementia and DLB—a process that appears to begin early in both conditions as evidenced by the findings in our MCI groups. Importantly, we show that impaired white matter microstructure of NBM pathways is more strongly associated with cognitive and attentional impairment than measures of NBM volume and might represent an early indicator of increased risk of conversion to dementia in people with MCI. These measures might therefore provide an additional marker of integrity of the cortical cholinergic system and have additional clinical utility as a progression predictor compared to NBM volume.

## Funding

This research was funded by Alzheimer’s Research UK (ARUK-PG2015-13) and a Wellcome Trust Intermediate Clinical Fellowship (WT088441MA) to J.P.T. and supported by the NIHR Newcastle Biomedical Research Centre (grant numbers BH120812 and BH120878) and Northumberland Tyne and Wear NHS Foundation Trust. G.E. Healthcare provided the FP-CIT radioligand for this investigator-led study.

## Competing interests

The authors report no competing interests.

## Supplementary material

Supplementary material is available at *Brain* online.

## Supplementary Material

awab372_Supplementary_Data

## Abbreviations

CAF: Clinician Assessment of Fluctuations
DLB: dementia with Lewy bodies
MCI: mild cognitive impairment
MCI-AD: mild cognitive impairment due to Alzheimer’s disease
MCI-LB: mild cognitive impairment with Lewy bodies
MMSE: Mini-Mental State Examination
NBM: nucleus basalis of Meynert
NPI: Neuropsychiatric Inventory
UPDRS: Unified Parkinson’s Disease Rating Scale
