# Supplementary material for: Cholinergic white matter pathways in dementia with Lewy bodies and Alzheimer’s disease
Source: Brain. 2021 Oct 4;145(5):1773–84. doi: 10.1093/brain/awab372 (PMC9166545; doi:10.1093/brain/awab372)
Supplement: awab372_Supplementary_Data [file awab372_supplementary_data.pdf]

Supplementary Table 1 Group comparison of radial and axial diffusivity. Mean (standard deviation) per diagnostic group and results from univariate ANCOVAs controlling for age, sex, and diffusivity from the white matter control mask. *P*-values from post-hoc tests are Bonferroni-corrected for multiple comparisons. All *P*-values from pairwise post-hoc comparisons that are not listed are >0.1

	Controls	MCI-AD	AD	MCI-LB	DLB	Group comparison
**Medial NBM pathway**						
Radial diffusivity	0.00062 (0.00005)	0.00065 (0.00007)	0.00067 (0.00006)	0.00063 (0.00005)	0.00065 (0.00006)	F(4,230)=1.4, *P*=0.24
Axial diffusivity	0.00132 (0.00005)	0.00135 (0.00007)	0.00138 (0.00007)	0.00135 (0.00005)	0.00137 (0.00007)	F(4,230)=8.5 *P*<0.001
						P(controls, AD)<0.001
						P(controls, DLB)=0.001
**Lateral NBM pathway**						
Radial diffusivity	0.00079 (0.00007)	0.00084 (0.0001)	0.00089 (0.00008)	0.00085 (0.00008)	0.00086 (0.0001)	F(4,230)=9.0, *P*<0.001
						P(controls, MCI-AD)=0.01
						P(controls, AD)<0.001
						P(controls, MCI-LB)<0.001
						P(controls, DLB)=0.003
Axial diffusivity	0.00133 (0.00008)	0.00138 (0.0001)	0.00144 (0.00008)	0.00139 (0.00008)	0.00140 (0.00009)	F(4,230)=13.0, *P*<0.001
						P(controls, MCI-AD)=0.07
						P(controls, AD)<0.001
						P(controls, MCI-LB)=0.003
						P(controls, DLB)<0.001

AD, Alzheimer's disease dementia; DLB, dementia with Lewy bodies; MCI-AD, mild cognitive impairment due to Alzheimer's disease; MCI-LB, MCI with Lewy bodies; NBM, nucleus basalis of Meynert
